# Expression and antiviral application of exogenous lectin (griffithsin) in sweetpotatoes

**DOI:** 10.3389/fpls.2024.1421244

**Published:** 2024-07-16

**Authors:** Shuai Liu, Yang Yu, Ke Guo, Qian Zhang, Zhaodong Jia, Morales Rodriguez Alfredo, Peiyong Ma, Hao Xie, Xiaofeng Bian

**Affiliations:** ^1^ Institute of Food Crops, Provincial Key Laboratory of Agrobiology, Jiangsu Academy of Agricultural Sciences, Nanjing, China; ^2^ Center for Tropical Crop Research, Research Institute of Tropical Roots and Tuber Crops (INIVIT), Santo Domingo, Cuba; ^3^ Xuzhou Institute of Agricultural Sciences, Chinese Academy of Agricultural Sciences, Xuzhou, China

**Keywords:** griffithsin, sweetpotato (*Ipomoea batatas* (L.) Lam), sweetpotato virus disease, sweetpotato leaf curl virus, antiviral genes

## Abstract

Griffithsin (GRFT) is a highly effective, broad-spectrum, safe, and stable viral inhibitor used to suppress a variety of viruses. However, little information is available on whether GRFT can prevent plant viral diseases. In this study, we constructed a GRFT overexpression vector containing the sweetpotato storage cell signal peptide and generated exogenous GRFT overexpression lines through genetic transformation. The transgenic plants showed notable resistance to sweetpotato virus disease in the virus nursery. To verify the antiplant virus function of GRFT, transient expression in tobacco leaves showed that GRFT inhibited the sweetpotato leaf curl virus (SPLCV). The replication of SPLCV was entirely inhibited when the concentration of GRFT reached a certain level. The results of pulldown and BIFC assays showed that GRFT did not interact with the six components of SPLCV. In addition, the mutated GRFT_D/A_ without the binding ability of carbohydrate and anticoronavirus function, in which three aspartate residues at carbohydrate binding sites were all mutated to alanine, also inhibited SPLCV. Quantitative reverse-transcription PCR analyses showed that the tobacco antiviral-related genes *HIN1*, *ICS1*, *WRKY40*, and *PR10* were overexpressed after GRFT/GRFT_D/A_ injection. Furthermore, *HIN1*, *ICS1*, and *PR10* were more highly expressed in the leaves injected with GRFT_D/A_. The results suggest that sweetpotato is able to express GRFT exogenously as a bioreactor. Moreover, exogenous GRFT expression inhibits plant viruses by promoting the expression of plant antiviral genes.

## 1 Introduction

Lectins comprise a class of proteins with a distinct molecular structure. The structure is specific and reversible to enable recognition of, and binding to, carbohydrates without altering the carbohydrate portion (Van Damme et al., 1998). Based on the recognition and binding properties of lectins for specific carbohydrates, lectins play important roles in signal transduction, immune response, and plant defense, including resistance to pests and antiviral diseases, and induction of apoptosis. At present, lectins have broad application prospects in insect control, disease resistance, the biopharmaceutical industry, and medical detection and therapy. Therefore, strategies to increase the yield of lectins are of practical importance.

Griffithsin (GRFT), a lectin with a high affinity for mannose residues derived from the marine algae *Griffithsia* spp., is a widely used antiviral inhibitor. The protein comprises 121 amino acids folded into stable domain-exchanged homomeric dimers and targets mannose-terminal residues of high manno-oligosaccharides. GRFT has a total of six carbohydrate binding sites per homologous dimer (Ziólkowska et al., 2006; Ziólkowska et al., 2007), which may be one reason why it is highly effective against various viruses, such as human immunodeficiency virus (HIV), hepatitis C virus (HCV), Japanese encephalitis virus, herpes simplex virus (HSV), and porcine reproductive and respiratory syndrome virus (Meuleman et al., 2012; Ishag et al., 2013; Takebe et al., 2013; Levendosky et al., 2015; Lusvarghi and Bewley, 2016; Millet et al., 2016; Derby et al., 2018; Li et al., 2018; Lee, 2019; Nabeta et al., 2021).

The mechanisms of action of GRFT against animal viruses are diverse. Among the aforementioned viruses, GRFT inactivates HIV almost immediately after exposure to the virus (Emau et al., 2007). First, GRFT inhibits the binding of HIV to CD4 on the surface of lymphocytes by selectively binding to the mannose-rich glycan gp120 on the viral envelope glycoprotein (Balzarini, 2005; Alexandre et al., 2010; Lee, 2019). In addition, GRFT can inhibit the binding of dendritic cells (DC-SIGN) to HIV-1, thereby preventing the virus from infecting target cells (Van Damme et al., 1998; Mori et al., 2005). GRFT acts at an early stage of the HCV life cycle, acting in a genotype-independent manner by intervening in the interaction between the viral envelope protein E2 and the viral receptor CD81 (Meuleman et al., 2012). In addition, GRFT inhibits the severe acute respiratory syndrome coronavirus (SARS-CoV), but, unlike the preceding two viruses, GRFT binds to the SARS-CoV surface glycoprotein S but does not affect it, and virus recognition of Vero cells is indicative of the receptor (Ziólkowska et al., 2006). GRFT can inhibit HSV-2 infection after entry by blocking cell-to-cell diffusion, but virus invasion of cells is not affected (Nixon et al., 2013). GRFT inhibits the internalization stage of the human papillomavirus (HPV) by binding to the HPV receptor integrin α6 (Levendosky et al., 2015; Derby et al., 2018). The most important antiviral mechanism of GRFT is to bind to glycoproteins on the surface of the capsular virus to inhibit virus transmission (O’Keefe et al., 2009).

GRFT has been expressed in several exogenous expression systems because of its low biotoxicity and extensive antiviral capacity, for example, an *Escherichia coli* expression system based on the pET expression vector (Giomarelli et al., 2006), a transient expression system in tobacco based on the tobacco mosaic virus (TMV) vector (O’Keefe et al., 2009; Fuqua et al., 2015a; Fuqua et al., 2015b; Vafaee and Alizadeh, 2018), a chloroplast expression system in tobacco (Hoelscher et al., 2018), and a rice endosperm expression system (Vamvaka et al., 2016). Lectins expressed in plants are observed to have stronger antiviral activity and no biological toxicity (O’Keefe et al., 2009). Thus, we speculated that GRFT can be directly expressed in feed crops as an additive to prevent the occurrence of animal capsular viroid diseases.

Compared with other crops, sweetpotato has the following advantages as a bioreactor. First, sweetpotato is a natural animal feed that does not require prior purification. Second, sweetpotato is a crop that reproduces asexually. Under suitable growing conditions, the aboveground biomass of sweetpotato provides a vegetable for humans as well as feed for livestock. Lastly, extraction of GRFT from sweetpotatoes in combination with the starch isolation process would reduce costs and pollution.

Sweetpotatoes could be used as a bioreactor to produce high-value GRFT protein or as an additive to animal feed to prevent the incidence of animal capsular viroid infection and thereby increase the utilization efficiency and economic benefits of sweetpotatoes. In this study, the sequence of GRFT was optimized according to the sequence preference for sweetpotatoes without altering GRFT dimer formation. For storage of GRFT in the vacuole of sweetpotato cells for easy extraction, the endoplasmic reticulum signal peptide and vacuole sorting signal sequences were inserted at either end of the GRFT sequence, and the His-tag protein motif for purification was added to the 3’ end of the GRFT sequence. The designed sequences were synthesized directly by the company and transformed under the control of different promoters in the vectors. Transgenic lines harboring GRFT were obtained successfully by genetic transformation. The transformants showed distinct resistance to sweetpotato virus disease (SPVD) in the field. SPVD is a viral disease that can cause a severe reduction in sweetpotato production. At present, there are few reports of lectins conferring resistance to plant viruses. Transient expression of GRFT in tobacco leaves inhibited the sweetpotato leaf curl virus (SPLCV). Bimolecular fluorescence complementation (BIFC) and pulldown analysis showed that GRFT did not interact with the virus. By constructing GRFT transgenic plants, we showed that GRFT-conferred resistance to plant viruses was not dependent on the carbohydrate-binding ability of the lectin. Quantitative reverse-transcription PCR (RT-qPCR) analysis revealed that the mechanism of GRFT resistance to plant viruses was to induce the expression of antiviral genes. The mechanism of GRFT against SPVD represents a novel strategy for plant virus control.

## 2 Materials and methods

### 2.1 Plant materials and growth conditions

The sweetpotato cultivar ‘Xushu 29’ was obtained from the Xuzhou Institute of Agricultural Sciences in Jiangsu, Xuhuai district, Xuzhou, Jiangsu, China. Plants were propagated from stem cuttings, and the potted plants were transferred to a clean greenhouse maintained at 28°C with a 16 h/8 h (light/dark) photoperiod. The sweetpotato plants were transplanted to the field for growth at a suitable temperature, and tubers were harvested in early November.

### 2.2 GRFT expression vector construction

The pCAMBIA1305–2×35S/Super/Sp/Swap4-GRFT vector was constructed. First, the pCAMBIA1305 vector was used as the basic expression vector, into which different promoter sequences (2×CaMV35S, Super, Sp, and Swap4) were inserted. Super promoter stems from the pCAMBIA-Super1300-GFP vector. The super promoter consists of a trimer of the octopine synthase transcriptional activating element affixed to the mannopine synthase2′ (mas2′) transcriptional activating element plus a minimal promoter. Sp (GC534664.1) is the sporamin gene promoter in sweetpotatoes. Swap4 promoter is a multistress-induced peroxidase promoter from sweetpotato. The fragment of the designed *GRFT* gene was synthesized by Bioengineer Biotechnology. The following primers to amplify the fragment were designed using the homologous recombination method: GRFT-SmaI(F): 5′-GtctcgaggaccggtcccgggATGAAGGCTTTCACTCTTGCCT-3′ and GRFT-PmlI(R): 5′-ctggtcaccaattcacacgtgATACTGCTCGTAATAGATATCAAGGGA-3′.

### 2.3 Sweetpotato transformation and positive transformant identification

Xushu 29 was used as the transgene recipient using the following transformation process. Sweetpotato stem tips were used for callus induction on MS1D medium (4.4 g/L Murashige and Skoog salts (MS), 0.4 mg/L thiamin, 30 g/L sucrose, 0.1 g/L inositol, and 1 mg/L 2,4-dichlorophenoxyacetic acid). In total, 10–15 ml of *Agrobacterium tumefaciens* strain EHA105 harboring the GRFT expression vector was gathered. The *Agrobacterium* cells were resuspended in MS1D supplemented with 100 μM acetosyringone (AS) and mixed with the sweetpotato calli. The mixture was shaken slightly for 30 min and then ultrasonicated for 10 s. Next, the calli were transferred to a solid medium (MS1D supplemented with 100 μM AS) with filter paper. After incubation in the dark for 2–3 days, the calli were washed with sterile water to remove the *Agrobacterium* cells and transferred to a screening medium (MS1D supplemented with 10 mg/L tauramycin and 400 mg/L cefotaxime). The calli were incubated under controlled conditions for 4–6 weeks. The calli were then transferred to an MSCH regeneration medium (4.4 g/L MS, 10 mg/L chaotropic acid, and 200 mg/L cefotaxime). After the shoots had differentiated from the calli, the shoots were transferred to SBMC rooting medium (4.4 g/L MS, 200 mg/L cefotaxime, and 0.3 mg/L thiamin). Genomic DNA was extracted from the plantlets for the identification of transformants.

### 2.4 Tobacco transient expression system

Tobacco seedlings were transferred to pots and cultivated in a greenhouse maintained at 28°C with a 16-h/8-h (light/dark) photoperiod for 1 month. The cell suspension of *Agrobacterium tumefaciens* strain GV3101 was shaken overnight (> 14 h) at 200–230 rpm at 28°C until the optical density (OD) value was 0.6–1.2. The cell suspensions were centrifuged at 1,000×*g* for 10 min. The collected cells were resuspended in MMA buffer (10 mM MgCl_2_, 10 mM MES, and 150 μM MAS, pH 5.6) and adjusted to OD = 1.00. Three leaves were injected with 1 ml of the *Agrobacterium* suspension using a 1-ml syringe, and the infection site was circled with a marker pen.

### 2.5 Sweetpotato protein extraction

Fresh sweetpotato leaf or root tissue (50 mg) was ground in liquid nitrogen into powder. The powdered samples were transferred to 1.5 ml centrifuge tubes, to which extraction buffer was added (50 mM Tris-HCl [pH 7.5], 5 mM EDTA, 10% glycerol, and 1% Triton-X-100). The mixture was placed on ice for 30 min, shaken every 10 min, then centrifuged at 12,000×*g* for 10 min at 4°C, and finally the supernatant was collected and stored at −20°C. Phenylmethylsulfonyl fluoride or other protease inhibitors were added before the extract was stored to prevent degradation.

### 2.6 Purification of tagged proteins and pulldown analysis

For His-tagged protein purification, the plasmid His::GRFT was transfected into *E. coli* strain BL21 (DE3) cells, and expression was induced by isopropyl β-d-1-thiogalactopyranoside (IPTG) by incubation for 12 h at 16°C. The complex proteins generated were purified using Ni-charged MagBeads (GenScript, L00295). For GST-tagged protein purification, the plasmid GST::GRFT was transfected into *E. coli* strain BL21 (DE3) cells, and protein expression was induced by IPTG by incubation for 12 h at 16°C. The GST-tagged proteins were purified using the GST-Sefinose Kit (Sangon Biotech, C600913, Shanghai, China). The eluted proteins were analyzed directly by SDS-PAGE or stored at −20°C.

### 2.7 Western blot analysis

The protein extracts with 5 × SDS-PAGE sample loading buffer were denatured in a boiling water bath for 10 min. Polyacrylamide gel electrophoresis was performed using 5%–10% SDS-PAGE gels for 1 h. The gels and alcohol-activated polyvinylidene fluoride (PVDF) membranes (Millipore) were immersed in the transfer solution for 10 min. Subsequently, the proteins were transferred from the gels to the PVDF membranes using a protein wet-transfer apparatus for 1.5 h at room temperature on a shaker using 5% skimmed milk. The primary antibodies anti-His (1:5,000, mouse, Abmart, M20020S, Shanghai, China), antiactin (1:5,000, mouse, TransGen Biotech, HC201–01, Beijing, China), and anti-GFP (1:5000, Rabbit, Abcam, EPR14104, Shanghai, China) were incubated overnight at 4°C on a shaker, where antiactin was used as an internal reference control. The secondary antibodies, comprising antimouse (1:10,000, Invitrogen, A11003, California, USA) and antirabbit (1:10,000, Invitrogen, A11008), were applied separately for binding to the primary antibody. The membranes were treated with a chemiluminescent solution (ZOMANBIO, ZD310, Beijing, China) and inspected with a chemiluminescence imager (Tanon, Shanghai, China).

### 2.8 Bimolecular fluorescence complementation analysis

The six components of SPLCV (AV1, AV2, AC1, AC2, AC3, and AC4) were linked to the vector PCV-NYFP. GRFT was linked to the vector PCV-CYFP. The NYFP-SPLCVs and CYFP-GRFT plasmids were transformed into *Agrobacterium* strain EHA105. Bimolecular fluorescence complementation analysis of the interaction between SPLCV and GRFT was assessed in the leaves of 3-week-old tobacco plants. The *Agrobacterium* strains harboring SPLCV-NYFP, GRFT-CYFP, and P19, respectively, were shaken overnight (> 14 h) at 200–230 rpm at 28°C. The suspensions of SPLCV-NYFP, GRFT-CYFP, and P19 were mixed in equal volumes. The mixture was injected into tobacco leaves, which were incubated for 3–4 days in the dark. The leaves were observed with a fluorescence microscope.

### 2.9 RNA extraction and RT-qPCR analysis

RNA extraction and quantitative real-time PCR analyses were performed as described previously (Bian et al., 2022). In brief, total RNA was extracted with the RNAprep Pure Plant Kit (polysaccharides and polyphenolics-rich) (Tiangen, Beijing, China). A sample (1 mg) of the purified total RNA was reverse-transcribed using the FastKing gDNA Dispelling RT SuperMix Kit (Tiangen). Primer pairs specific to the tested genes were designed using the NCBI Primer-BLAST online tool. The qRT-PCR analysis was performed using a SYBR^®^ Premix Ex Taq™ Kit (TaKaRa, Beijing, China) on an ABI Prism^®^ 7900 Real-Time PCR System. The *Nicotiana benthamiana* Ubiquitin C (*UBC*) gene (GenBank: AB026056.1) was used as an internal reference. Relative changes in gene expression were calculated using the 2^−ΔΔCt^ method. Primers used for qPCR analyses are listed in **Supplementary Table S1**.

## 3 Results

### 3.1 Construction of the GRFT gene expression vector

**Figure 1:**
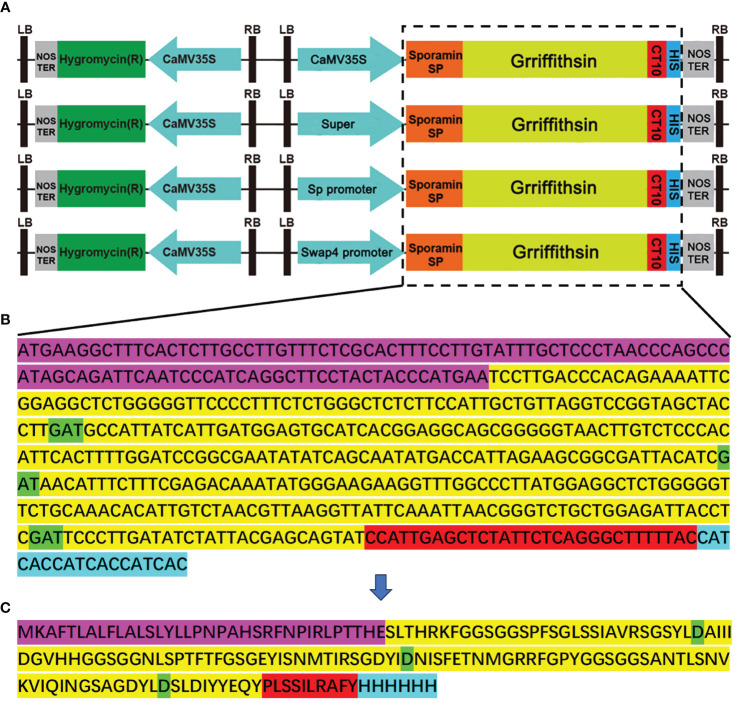
Construction of GRFT overexpression vectors. The pCambia1305-GFP was used as the skeleton to construct the vectors. **(A)** Linear vector map of different promoters activated. **(B)** Nucleic acid sequence of modified GRFT. **(C)** The amino acid sequence of GRFT. The purple-highlighted sequence is the sweetpotato endoplasmic reticulum signaling peptide. The yellow-highlighted sequence is GRFT. The green-highlighted aspartic acid residues are crucial sites for carbohydrate binding. The red-highlighted sequence is the sweetpotato vacuole sorting signal. The blue-highlighted sequence is the His-tag.

The monomer GRFT is composed of 121 amino acids, based on the complete sequence lodged in the GenBank database (accession no. FJ594069). According to the preferred plant base sequence, the sequences of GRFT were optimized without changing the amino acid sequence. Each GRFT monomer has three carbohydrate-binding sites, namely, D30, D70, and D112 (Xue et al., 2013). To improve protein stability, the GRFT protein was targeted for storage in vacuoles by inserting the endoplasmic reticulum signal peptide sequence at the N terminus and the vacuole sorting signal sequence at the C-terminus. For easy detection, the His-tag protein motif was added to the 3’ end of the GRFT sequence. The final sequence (516 bp, 172 aa) was driven by the promoters CaMV35S, a Super promoter, or a sweetpotato promoter (Sp or Swap4) (**Figure 1**). **Figure 1A** shows the linear vector map of different promoters activated. The final nucleic acid sequence and amino acid sequence are shown in **Figures 1B, C**, respectively.

### 3.2 Confirmation of sweetpotato transformants expressing GRFT

**Figure 2:**
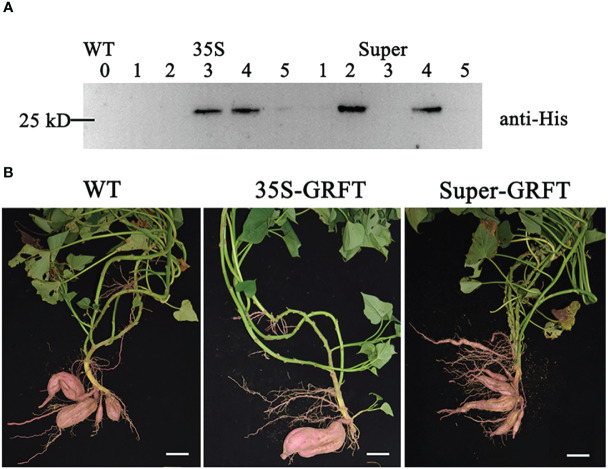
Sweetpotato transgenic lines expressing GRFT. The wild type (WT) was ‘Xushu 29’; 35S-GRFT and Super-GRFT are transgenic lines with GRFT driven by different promoters. **(A)** WT and transgenic lines were screened by Western blotting. **(B)** Tubers of the WT and transgenic lines after harvest. Scale bar: 5 cm.

The constructed vectors and empty vector (WT) were genetically transformed into the sweetpotato cultivar ‘Xushu 29’. Through hygromycin screening, the verified transgenic plants expressed GRFT driven by the CaMV35S and Super promoters rather than Sp or Swap4. Subsequently, the transgenic lines stably expressing GRFT were screened by western blot analysis with the anti-His antibody (**Figure 2A**). Owing to amino acids 15–19, GRFT usually exists as a homologous dimer, which is the optimal state for its antiviral function (Xue et al., 2013). In this study, four transformants were identified with bands larger than 25 kD, which is approximately twice the molecular weight of the GRFT monomer (12.7 kD). Therefore, the optimization of the *GRFT* sequence did not affect the structure of the GRFT protein. The transgenic lines expressing GRFT developed harvestable tubers similar to those of the wild type (**Figure 2B**).

### 3.3 Identification of the antiplant virus function of GRFT

**Figure 3:**
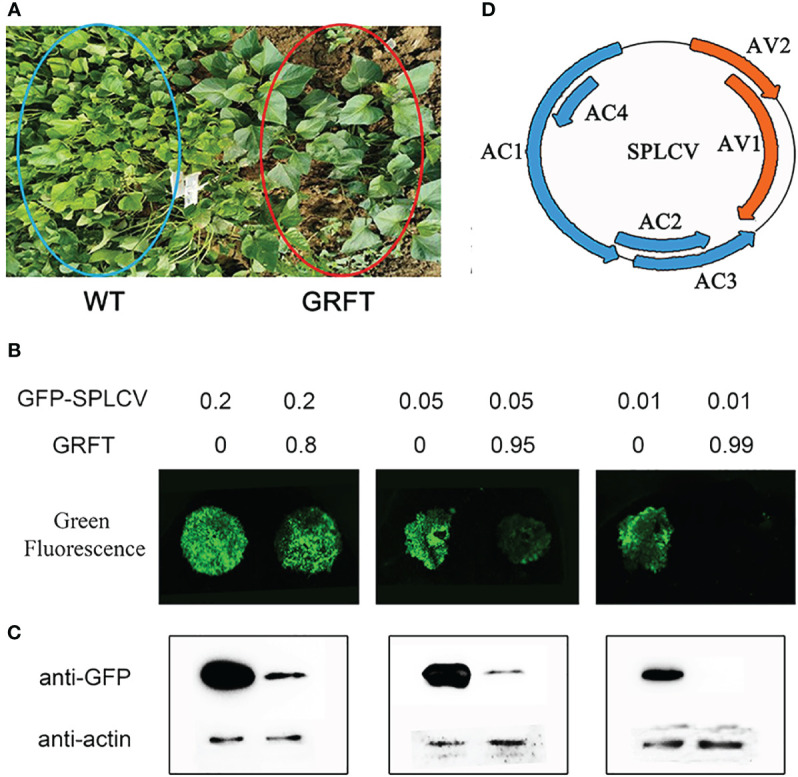
Resistance to plant viruses conferred by GRFT. **(A)** Transgenic lines were grown in the virus nursery for SPVD outbreaks. **(B)** Transient expression of GFP-SPLCV and 35S-GRFT in tobacco leaves. The infection solution (concentration OD = 1) was mixed in different proportions. **(C)** After transient expression, the protein was extracted from the tobacco leaves for Western blotting analysis. **(D)** The six components of SPLCV.

It is well known that the anti-animal virus function of lectins is mainly affected by their binding to viral surface glycoproteins. Plant viruses lack glycoproteins, and currently, there are few reports of lectins effective against plant viruses. Interestingly, the transgenic plants expressing GRFT showed obvious resistance to SPVD in the virus disease nursery (**Figure 3A**). SPVD is a contagious viral disease caused by coinfection with the sweetpotato feather mottle virus (SPFMV) and the sweetpotato chlorotic stunt virus (SPCSV). Almost all sweetpotato plants in the virus nursery were infected with SPVD, except for GRFT-transgenic plants. Therefore, we speculated that GRFT may provide broad-spectrum resistance to plant viruses. SPLCV is a single-component bipartite virus that can infect a variety of dicotyledonous plants, including sweetpotato, tobacco, and petunia. To test this hypothesis, CaMV35S:GRFT and CaMV35S:SPLCV-GFP vectors were transfected via *Agrobacterium tumefaciens* strain GV3101 and used to simultaneously infect tobacco leaves according to different ratios (**Figure 3B**). At a ratio of 4:1, the green fluorescence signal was slightly weaker than that observed after injection of the same concentration of the viral expression vector. When the relative concentration of GRFT was 19 times that of SPLCV-GFP, the green fluorescence signal was weak. No green fluorescence was visible when the concentration of GRFT was 99 times that of SPLCV-GFP (**Figure 3B**). The protein at the infection site was extracted for western blot analysis with the anti-GFP antibody. The results showed that the GFP signal gradually weakened with the increase in GRFT proportion (**Figure 3C**). Thus, the results of transient expression of GRFT in tobacco were consistent with the western blotting results. This phenomenon suggests that GRFT can inhibit infection by SPLCV and that GRFT may provide broad-spectrum resistance to plant viruses.

### 3.4 Mechanism of GRFT resistance to plant viruses

**Figure 4:**
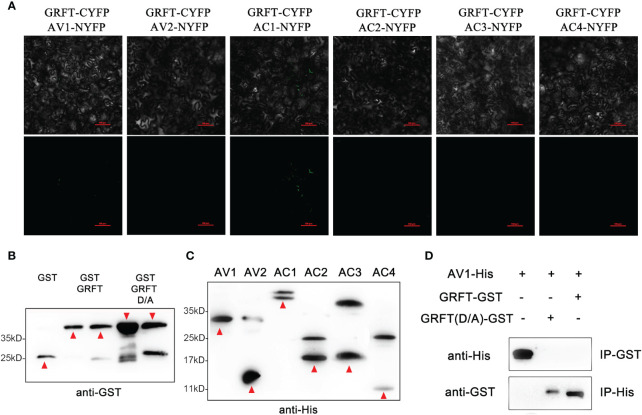
Interaction between GRFT and SPLCV verified by BIFC and pulldown assays. **(A)** Bimolecular fluorescence complementation assay revealing noninteraction between GRFT and SPLCVs. SPLCVs and GRFT were fused to the N- or C-terminal portion of YFP, and the fusion proteins were co-expressed in *Nicotiana benthamiana* leaves. Images were obtained using a confocal laser-scanning microscope at 3 dpi. The experiment was performed three times with similar results. Scale bars: 10 μm. **(B)** Purified His-tagged proteins GST-GRFT and GST-GRFT_D/A_ were analyzed by Western blotting with the anti-GST antibody. Red arrowheads point to the correct protein band. **(C)** Purified His-tagged proteins His-SPLCVs (AV1–AC4) were analyzed by Western blotting with the anti-His antibody. Red arrowheads point to the correct protein band. **(D)** SPLCVs and GRFT do not interact *in vitro*. GST-GRFT or GST-GRFT_D/A_ were immobilized on glutathione sepharose beads, incubated with the His-AV1(CP) protein, and subjected to immunoblot analysis with an anti-His antibody. The His-AV1(CP) protein was immobilized on Ni-charged MagBeads, incubated with GST-GRFT or GST-GRFT_D/A_, and subjected to immunoblot analysis with an anti-GST antibody. The results for the other components of SPLCV were consistent and were not shown.

**Figure 5:**
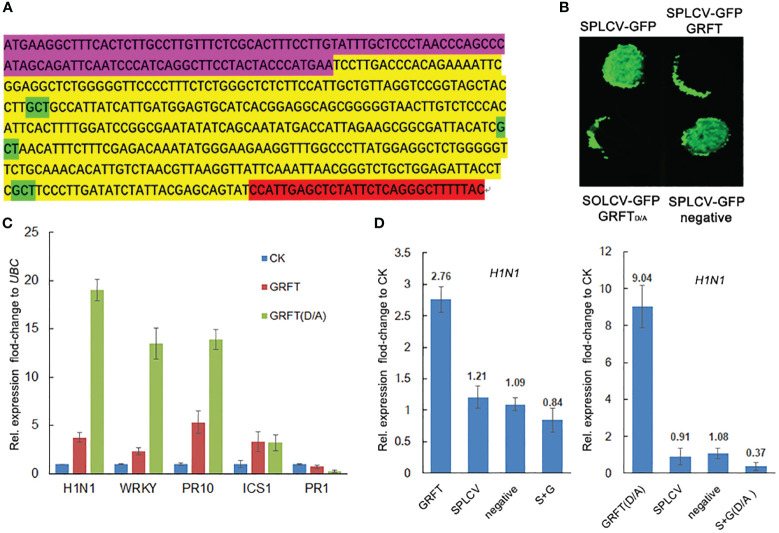
Mechanism of inhibition of SPLCV by GRFT. **(A)** Three aspartate residues of GRFT were replaced by alanine to generate GRFT_D/A_. **(B)** Four combinations of constructs were transiently expressed in the leaves of *Nicotiana benthamiana*, namely, SPLCV-GFP, SPLCV-GFP+GRFT, SPLCV-GFP+GRFT_D/A_, and SPLCV-GFP+negative. The infection solution (concentration OD = 0.5) was mixed. Negative is the empty 35S vector. **(C)** Expression of antiviral genes detected using qRT-PCR in *N. benthamiana* leaves infected with GRFT or GRFT_D/A_ for 3 days. CK is untreated leaves. Bars and error bars represent the mean and standard error of the fold-change in expression level relative to *UBC* calculated from three independent experiments each with three biological replicates. **(D)** *H1N1* expression in *N. benthamiana* leaves infected with GRFT, SPLCV, negative, or GRFT+SPLCV (or GRFT_D/A_+SPLCV). Bars and error bars represent the mean and standard error of the fold-change in expression level relative to the CK calculated from three independent experiments each with three biological replicates. CK is untreated leaves. Statistical significance in **(C, D)** was assessed using one-way ANOVA followed by Tukey’s test.

GRFT is known to provide resistance to coronaviruses by binding to the surface coat protein of the virus, thus inhibiting the transmission of the virus. The shells of plant viruses lack glycoproteins, so GRFT may inhibit plant viruses by binding to other components of plant viruses. The SPLCV genome is approximately 2.8 kb in length and consists of six open reading frames (**Figure 3D**). The six components are AV1 (29.36 kD), AV2 (13.34 kD), AC1 (40.88 kD), AC2 (16.60 kD), AC3 (16.70 kD), and AC4 (9.48 kD), of which AV1 is the coat protein (CP). The pCMV-C-YFP vectors associated with these six components were coinjected into *Nicotiana benthamiana* leaves with pCMV-N-YFP : GRFT. No fluorescence signal was observed (**Figure 4A**). These results suggested that GRFT does not bind to SPLCV. To verify this conclusion, GST-GRFT and His-tagged SPLCV components were purified for pulldown *in vitro* (**Figures 4B, C**). Similarly, no component of SPLCV was combined with GRFT (**Figure 4D**). In conclusion, GRFT was indicated to combat plant viruses by a different mechanism than how it inhibits animal viruses. Three aspartic acid residues associated with sugar binding in GRFT were simultaneously point-mutated to alanine (**Figure 5A**). GRFT_D30,70,112A_ with the His-tag and signal peptide was attached to the CaMV35S vector. GRFT_D/A_ inhibited SPLCV expression as effectively as GRFT in the tobacco leaf transient expression experiments (**Figure 5B**). SPLCV was not suppressed by the negative control (the empty CaMV35S vector). Similarly, GRFT_D/A_ did not interact with SPLCV (**Figure 4D**). These results further indicated that the antiviral function of GRFT in plants was independent of its sugar-binding activity.

### 3.5 Expression of antiviral genes in tobacco was induced by GRFT

Given that GRFT did not bind to SPLCV, we hypothesized that GRFT induced the expression of antiviral genes in the plant. To date, many genes with antiviral functions have been identified in tobacco, such as hairpin-induced 1 (*HIN1*), isochorismate synthase I (*ICS1*), *WRKY40*, pathogenesis-related 1 (*PR1*), and *PR10* (van Verk et al., 2011; Hao et al., 2018; Peng et al., 2019; Jiang et al., 2021). The expression of these genes was examined in tobacco leaves injected with 35S:GRFT or 35S:GRFT_D/A_ or no construct (CK). Compared with the CK, the expression levels of *HIN1*, *ICS1*, *WRKY40*, and *PR10* in leaves infected by GRFT or GRFT_D/A_ were significantly increased. The changes in expression of *HIN1*, *WRKY40*, and *PR10* caused by GRFT_D/A_ were more strongly significant (**Figure 5C**). 35S:GRFT/GRFT_D/A_, SPLCV, the negative control (empty vector), and both constructs (35S:GRFT/GRFT_D/A_+SPLCV) were injected separately on the same leaf. 35S:GRFT increased *HIN1* expression; 35S:GRFT_D/A_ had a stronger effect; but SPLCV did not cause any change in expression. It was interesting that the expression of *H1N1* decreased in leaves injected with both GRFT and SPLCV (**Figure 5D**). This phenomenon suggests that GRFT may inhibit SPLCV by inducing the expression of antiviral genes.

## 4 Discussion

### 4.1 Sweetpotato is suitable as a bioreactor for the exogenous expression of GRFT

GRFT is an efficient, broad-spectrum, safe, and stable anti-animal virus inhibitor and is currently the main lectin candidate drug used in the clinical prevention of a variety of viruses (Barton et al., 2014; Derby et al., 2018). Therefore, establishing a stable and high-yielding GRFT expression system is important. At present, researchers have experimented with a variety of exogenous expression systems, such as *E. coli* (Giomarelli et al., 2006), tobacco (Hoelscher et al., 2018), and rice (Vamvaka et al., 2016). However, exogenous expression of GRFT in sweetpotatoes is advantageous. First, the tubers and leaves of sweetpotatoes can be directly utilized as natural feed for livestock, which is not possible without the processing of rice. The use of sweetpotato-expressing GRFT as a feed additive will help to prevent the incidence of animal capsular viroid diseases. Second, sweetpotatoes can be continuously grown under suitable greenhouse conditions, providing fresh material for GRFT extraction at any time. In the present study, we successfully obtained GRFT transgenic lines expressing storage cell signaling peptides. In the following study, chloroplast expression systems and other promoters will be attempted to generate sweetpotato transgenic lines with a higher GRFT content. The use of sweetpotatoes as a bioreactor will be of considerable importance to increasing the yield of GRFT.

### 4.2 GRFT functions in resistance to plant viruses

As devastating plant pathogens, plant viruses pose a serious threat to global food security and economic development (Savary et al., 2019; He and Krainer, 2020). To date, more than 2,000 different types of plant viruses have been reported, and, with the rapid development of virus identification and diagnostic technologies, this number is expected to continue to increase. Research on resistance to plant viruses has mainly investigated three aspects: autophagy, plant hormones, and resistance genes. Autophagy is a process in which eucaryotes perform the turnover of intracellular substances. Through autophagy, plants can “consume” viruses (Yang and Liu, 2022). In recent years, the glycoprotein of the rice stripe mosaic virus was found to directly interact with OsSnRK1B, thus activating autophagy and hindering virus proliferation (Huang et al., 2024). Similarly, C1 proteins from the Tomato Frizzy-Yunnan virus and several other geminiviridaes have been targeted for autophagy degradation through interaction with the ATG8h protein (Li et al., 2022). Plant hormones and their signaling regulatory networks play an important role in regulating plant–virus interactions (Alazem and Lin, 2015; Zhao and Li, 2021). Li et al. revealed that the jasmonic acid signaling pathway in rice can promote the immune mechanism against the rice stripe virus (RSV) virus by regulating RNA silencing. Interestingly, rice plants infected with RSV show abscisic acid accumulation, which further promotes viral infection (Cui et al., 2021). Nucleotide oligomerization and binding domain-like receptors (NLRs) are typical antiviral genes in many plant species, effective against soybean mosaic virus, potato virus Y (PVY), and TMV (Yin et al., 2021). The first monocotyledon NLR gene to be cloned was from *Brachypodium* (Wu et al., 2022). Phytolectins are widely used for their antiviral properties, but this function has rarely been reported in plants. This is mainly because the antiviral function of lectins is achieved through their specific binding to viral surface carbohydrates, whereas plant viruses lack envelope structures containing glycoproteins. Nevertheless, some studies of lectins inhibiting plant viruses have been reported. For example, at certain concentrations, poplar mushroom lectin can inhibit TMV (Sun et al., 2003). Certain mushroom lectins can inhibit plant viruses, such as *Paxillus involutus* lectins, which reduce the toxicity of TMV (Peumans and Vandamme, 1995). However, the mechanism of lectin-conferred resistance to plant viruses has not been elucidated previously. In the current study, exogenous expression of GRFT in sweetpotatoes inhibited SPVD infection. In addition, GRFT inhibited SPLCV expression. SPVD is caused by two RNA viruses, namely, SPFMV and SPSCV. SPLCV is a DNA virus. Therefore, we speculate that GRFT also has a broad-spectrum antiviral function in plants. Confirmation of this conclusion will provide a novel strategy for future plant antiviral research.

### 4.3 Mechanism of GRFT resistance to plant viruses

Plant viruses lack envelope proteins that can interact with GRFT but do have coat proteins that control viral proliferation. In the present study, BIFC and pulldown assays determined that GRFT did not interact with the six components of SPLCV, including the coat protein (AV2). Further investigation showed that the simultaneous site-specific mutation of three important sugar-binding sites of GRFT did not influence its antiviral effect. These results suggested that GRFT resistance to plant viruses does not depend on its ability to bind to carbohydrates. In recent years, certain studies have reported that lectins can interact with glycoproteins or extracellular glycans on the cell wall to indirectly resist infection by plant viruses. Ayouba et al. (Ayouba et al., 1994) point out that the seed lectins of several legumes can strongly interact with cell wall peptide polymers. Lectins extracted from potatoes can distinguish between non-toxic and toxic strains of *Raleella soliculosa* and prevent the toxic strains from being adsorbed on the cell wall. Other lectins block the spread of viruses by killing the pests that transmit them. *Galanthus nivalis* agglutinin has a strong toxic effect on rice brown planthoppers and black-tailed leafhoppers and can also inhibit the growth of aphids (Du et al., 2000; Wang et al., 2005; Michiels et al., 2010). Neither the interaction with the cell wall nor the killing of pests is consistent with the mechanism of GRFT resistance against plant viruses. Therefore, we speculate that GRFT may induce the expression of antiviral genes in plants. To date, many genes with antiviral functions have been mined in plants. For example, *NbHIN1* expression induced by exogenous Harpin protein induces allergic reactions in plants, and its overexpression in tobacco inhibits TMV (Peng et al., 2019). Overexpression of *NbWRKY40* increased resistance to tomato mosaic virus (ToMV), whereas knockdown of *NbWRKY40* increased the susceptibility of *NbWRKY*-silenced plants to ToMV infection (Jiang et al., 2021). *ICS1* promotes salicylic acid production after a tobacco infection with the virus. Pathogenesis-related gene expression can be induced by SA (Baebler et al., 2014; Liang et al., 2024). Similarly, in *Arabidopsis thaliana*, vanisulfane can trigger salicylic acid accumulation, upregulate the expression of *ICS1* and *PR1*, and induce resistance to PVY in transgenic *Arabidopsis* (Zhang et al., 2023). Treatment of Arabidopsis leaves with benzazol-*S*-methyl acid significantly increased the expression of the systemic acquired resistance-related genes *PR1*, *SID2*, and *ALD1* and inhibited Plantago mosaic virus infection after 1 day (Ito et al., 2024). In the present study, the expression levels of the antiviral genes *HIN1*, *ICS1*, *WRKY40*, and *PR10* were significantly increased in tobacco 3 days after GRFT injection. Therefore, we hypothesized that exogenous GRFT resistance to plant viruses is induced by the expression of plant antiviral genes. Interestingly, the expression levels of *HIN1*, *WRKY40*, and *PR10* were higher in tobacco injected with GRFT_D/A_. This finding suggests that the ability of GRFT to bind to carbohydrates is an inhibitor, rather than a booster, of resistance to plant viruses. Confusingly, in tobacco leaves injected with both GRFT and SPLCV, the expression of *H1N1* was reduced. We hypothesize that *H1N1* is not a major anti-SPLCV gene. GRFT may induce a major anti-SPLCV protein product, which inhibits the expression of the virus and *H1N1* by interacting with SPLCV. Because GRFT can induce additional antiviral substances, the simultaneous injection of GRFT mutations and SPLCV makes the inhibition of *H1N1* more pronounced. In summary, the present results imply that exogenous expression of GRFT can lead to resistance to plant viral diseases by inducing the expression of endogenous antiviral genes in the plant.

## Data availability statement

The original contributions presented in the study are included in the article/**Supplementary Material**. Further inquiries can be directed to the corresponding author.

## Author contributions

SL: Conceptualization, Data curation, Formal Analysis, Funding acquisition, Investigation, Methodology, Software, Validation, Visualization, Writing – original draft, Writing – review & editing. YY: Data curation, Writing – review & editing, Funding acquisition, Methodology, Software. KG: Writing – review & editing, Data curation, Formal Analysis. QZ: Investigation, Writing – review & editing. ZJ: Writing – review & editing, Formal Analysis, Methodology, Software. MA: Writing – review & editing, Investigation, Funding acquisition. PM: Writing – review & editing, Methodology. HX: Data curation, Writing – review & editing. XB: Conceptualization, Data curation, Formal Analysis, Funding acquisition, Project administration, Resources, Supervision, Validation, Writing – original draft, Writing – review & editing.
