# Supplementary material for: Expression and antiviral application of exogenous lectin (griffithsin) in sweetpotatoes
Source: Front Plant Sci. 2024 Jul 16;15:1421244. doi: 10.3389/fpls.2024.1421244 (PMC11286482; doi:10.3389/fpls.2024.1421244)
Supplement: Supplementary file 1 [file Table_1.docx]

Supplementary Material

# Supplementary Table

Supplement Table S1 Primer pairs sequence.

| PCR identification of transgenic plants | |
| --- | --- |
| *Hyg-F* | ACACAGCCATCGGTCCAGAC |
| *Hyg-R* | ATCTTAGCCAGACGAGCGGG |
| pulldown Vector construction Primers | |
| GST-GRFT-F | ttccaggggcccctgggatccTCCTTGACCCACAGAAAATTCG |
| GST-GRFT-R | ctcgagtcgacccgggaattcCTAATACTGCTCGTAATAGATATCAAGGG |
| His-AV1-F | cagcaaatgggtcgcggatccATGACAGGGCGAATTCCCG |
| His-AV1-R | ttgtcgacggagctcgaattcATTATTATGTGAATCATAGAAATAAGCCC |
| His-AV2-F | cagcaaatgggtcgcggatccATGGATACCCTGTGGGACCC |
| His-AV2-R | ttgtcgacggagctcgaattcCCCTTCCTTCTCTTCATCCGG |
| His-AC1-F | cagcaaatgggtcgcggatccATGCCTCGTCAAGCAAGTTTCC |
| His-AC1-R | ttgtcgacggagctcgaattcGGAACTTTCCTCTTGGCCTTCT |
| His-AC2-F | cagcaaatgggtcgcggatccATGTCCAATCTCCCTTCTGGAA |
| His-AC2-R | ttgtcgacggagctcgaattcAGGCGTTCCAAAATACCAGTCC |
| His-AC3-F | cagcaaatgggtcgcggatccATGGATTCACGCACAGGGG |
| His-AC3-R | ttgtcgacggagctcgaattcATACAGTAATACCTTTACATCATCATTACAATC |
| His-AC4-F | cagcaaatgggtcgcggatccATGGGTCTCTGCACCTCCATG |
| His-AC4-R | ttgtcgacggagctcgaattcAGGCTTCTGCTGCTGCATCA |
| BIFC Vector construction Primers | |
| BIFC-NF | GACGTAAGGGATGACGCACA |
| BIFC-NR | GCTGAACTTGTGGCCGTTTA |
| BIFC-CF | GGATGACGCACAATCCCACT |
| BIFC-CR | GTTCTGCTGGTAGTGGTCGG |
| BIFC-GRFT-F | cgagctcggtacccgggatccATGAAGGCTTTCACTCTTGCCT |
| BIFC-GRFT-R | cgcgtacgagatctggtcgacGTGATGGTGATGGTGATGGTAAAA |
| BIFC-AV1-F | ccggggcggtacccgggatccATGACAGGGCGAATTCCCG |
| BIFC-AV1-R | tgtagtccatttgttggatccTTAATTATTATGTGAATCATAGAAATAAGCC |
| BIFC-AV2-F | ccggggcggtacccgggatccATGGATACCCTGTGGGACCC |
| BIFC-AV2-R | tgtagtccatttgttggatccTCACCCTTCCTTCTCTTCATCCG |
| BIFC-AC1-F | ccggggcggtacccgggatccATGCCTCGTCAAGCAAGTTTCC |
| BIFC-AC1-R | tgtagtccatttgttggatccTCAGGAACTTTCCTCTTGGCC |
| BIFC-AC2-F | ccggggcggtacccgggatccATGTCCAATCTCCCTTCTGGAA |
| BIFC-AC2-R | tgtagtccatttgttggatccTTAAGGCGTTCCAAAATACCAGT |
| BIFC-AC3-F | ccggggcggtacccgggatccATGGATTCACGCACAGGGG |
| BIFC-AC3-R | tgtagtccatttgttggatccTTAATACAGTAATACCTTTACATCATCATTACA |
| BIFC-AC4-F | ccggggcggtacccgggatccATGGGTCTCTGCACCTCCATG |
| BIFC-AC4-R | tgtagtccatttgttggatccTTAAGGCTTCTGCTGCTGCAT |
| Quantitative PCR Primers | |
| NbPR10-qF | GGGGTGCTGGAAGCATAAAA |
| NbPR10-qR | CGAACTCAAGTTTGTCGCCT |
| NbPR1-qF | GTGCCCAAAATTCTCAACA |
| NbPR1-qR | AAATCGCCACTTCCCTCAG |
| NbWRKY-qF | ACTCCCTCCGATCAAAGTGC |
| NbWRKY-qR | GGCACGATGGTGCAAAAGAA |
| HIN1-qF | TTCCGCCACCAGCAAAATC |
| HIN1-qR | TTAGGACGAAGAACGAGCCATA |
| NbICS-qF | TCATCACTCGTGAAATGGTCG |
| NbICS-qR | GAGGCTGGGAGTTAACCAAGT |
| NbUBC-qF | TGGAGGTACATTTAAGCTGACAC |
| NbUBC-qR | TCACAGAGCAAAGACTGGATTG |
| Nbactin-qF | CTTGAAACAGCAAAGACCAGC |
| Nbactin-qR | GGAATCTCTCAGCACCAATGG |
